# Systematic Review on COVID-19 Readmission and Risk Factors: Future of Machine Learning in COVID-19 Readmission Studies

**DOI:** 10.3389/fpubh.2022.898254

**Published:** 2022-05-23

**Authors:** Wei Kit Loo, Khairunnisa Hasikin, Anwar Suhaimi, Por Lip Yee, Kareen Teo, Kaijian Xia, Pengjiang Qian, Yizhang Jiang, Yuanpeng Zhang, Samiappan Dhanalakshmi, Muhammad Mokhzaini Azizan, Khin Wee Lai

**Affiliations:** ^1^Department of Biomedical Engineering, Faculty of Engineering, Universiti Malaya, Kuala Lumpur, Malaysia; ^2^Department of Rehabilitation Medicine, Faculty of Medicine, Universiti Malaya, Kuala Lumpur, Malaysia; ^3^Department of Computer System and Technology, Faculty of Computer Science and Information Technology, Universiti Malaya, Kuala Lumpur, Malaysia; ^4^School of Artificial Intelligence and Computer Science, Jiangnan University, Wuxi, China; ^5^Department of Medical Informatics of Medical (Nursing) School, Nantong University, Nantong, China; ^6^Department of ECE, Faculty of Engineering and Technology, SRM Institute of Science and Technology, Kattankulathur, India; ^7^Department of Electrical and Electronic Engineering, Faculty of Engineering and Built Environment, Universiti Sains Islam Malaysia, Nilai, Malaysia

**Keywords:** COVID-19, readmission, risk factors, mortality, machine learning

## Abstract

In this review, current studies on hospital readmission due to infection of COVID-19 were discussed, compared, and further evaluated in order to understand the current trends and progress in mitigation of hospital readmissions due to COVID-19. Boolean expression of (“COVID-19” OR “covid19” OR “covid” OR “coronavirus” OR “Sars-CoV-2”) AND (“readmission” OR “re-admission” OR “rehospitalization” OR “rehospitalization”) were used in five databases, namely Web of Science, Medline, Science Direct, Google Scholar and Scopus. From the search, a total of 253 articles were screened down to 26 articles. In overall, most of the research focus on readmission rates than mortality rate. On the readmission rate, the lowest is 4.2% by Ramos-Martínez et al. from Spain, and the highest is 19.9% by Donnelly et al. from the United States. Most of the research (*n* = 13) uses an inferential statistical approach in their studies, while only one uses a machine learning approach. The data size ranges from 79 to 126,137. However, there is no specific guide to set the most suitable data size for one research, and all results cannot be compared in terms of accuracy, as all research is regional studies and do not involve data from the multi region. The logistic regression is prevalent in the research on risk factors of readmission post-COVID-19 admission, despite each of the research coming out with different outcomes. From the word cloud, age is the most dominant risk factor of readmission, followed by diabetes, high length of stay, COPD, CKD, liver disease, metastatic disease, and CAD. A few future research directions has been proposed, including the utilization of machine learning in statistical analysis, investigation on dominant risk factors, experimental design on interventions to curb dominant risk factors and increase the scale of data collection from single centered to multi centered.

## Introduction

### Overview of COVID-19

COVID-19 is an infectious disease caused by a novel coronavirus named 2019-nCoV by WHO (1). The virus can spread from person to person through respiratory droplets and close contact. Some common symptoms that can be observed include dry cough, fever, and tiredness. Some patients may experience shortness of breath, body aches, and pains, nasal congestion, runny nose, sore throat, or diarrhea. The incubation period of COVID-19 can last for 2 weeks or longer. The disease may still be infectious even during the period of latent infection.

Till 18 December 2021, a total of more than 271 million cases of Covid 19 have been reported to World Health Organization (WHO) worldwide, with a total of more than 5.3 million of deaths (2). Of the reported cases, many of them were readmitted, and a percentage of them were dead subsequently. According to research on the nationwide Veteran Affairs healthcare system, within 60 days of discharge, 19.9% who survived COVID-19 hospitalization were readmitted with 9.1% death (3). In addition, research from Einstein Medical Center Philadelphia found that 7.6% of patients were readmitted within 1 month, while 24% were readmitted within 6 months of the initial hospitalization with 9% readmitted patient death (4). From the research statistics, the percentage of readmission due to COVID-19 is quite high with the percentage around 20%.

The ways that COVID-19 affects the body seem to get mysterious due to the continuity of mutated viral variants development. Many patients' symptoms fully disappear before they suddenly and unexpectedly begin deteriorating. Other patients have recovered and tested “negative” but later tested “positive” again. While the medical community is still struggling to fully understand the novel coronavirus that causes COVID-19, there appears to be an emerging concern that virus reinfection has been reported in recovered or even vaccinated patients. This raises new questions among scientists and healthcare authorities after the country successfully flattened the curve. There is no clear evidence of what causes the reinfection after recovery and vaccination.

Since the outbreak was in 2019, the public has mostly focused on the direct and indirect effects of COVID-19. Studies of the long-term effects on long covid and its associated rehospitalization are needed to effectively plan healthcare delivery and capacity. Despite the long-term consequences on public health becoming clearer from time to time, an investigation on factors that lead to rehospitalization is urgently needed.

### Reinfection and Recurrence of COVID-19

Reinfection is the infection following recovery from or superimposed on an infection of the same type (5), in this case, if the patient is infected more than one time of COVID-19, then he is said to have reinfection.

On the other hand, recurrence is the new occurrence of something that happened or appeared before (6). To be more precise, recurrence of COVID-19 is the repeated occurrence of morbidities or symptoms due to previous SARS-CoV-2 infection.

### Hospital Readmission and Its Impact on the Society

The reinfection and recurrence of COVID-19 may be directly impacting the hospital readmission rate, despite the comorbidities being an external factor contributing to the readmission as well. Looking into its significance, as readmissions may further pull resources from an already overwhelmed healthcare system, such as the issue of bed productivity. As a long-term consequence, readmission will waste a lot of public resources and it may cause new waves of the covid pandemic (7). This will lead to more secondary damage to the country economically, including an increase in job unemployment and a declination of GDP. Outcomes on long-term outcomes could be a reference for physicians and even policy makers in making decisions on discharge, hospital-capacity planning, and possibly patient monitoring after discharge for patients with COVID-19. Readmission rate is also an important parameter of patient safety and a hospital's quality performance.

### Work Motivation and Objective of Systematic Review

As mentioned earlier, unplanned or unwanted hospital readmissions create a burden on society while the long-term consequences post COVID-19 infection remain uncertain to the public. In this review, current studies on hospital readmission due to infection of COVID-19 were discussed, compared, and further evaluated in order to understand the current trends and progress in the mitigation of hospital readmissions due to COVID-19. At the same time, challenges faced, a gap of knowledge and potential research directions were discussed to provide insights for future research.

### Search Strategy and Eligibility Criteria

Boolean expression of (“COVID-19” OR “covid19” OR “covid” OR “coronavirus” OR “sars-cov-2”) AND (“readmission” OR “re-admission” OR “rehospitalization” OR “rehospitalization”) were used in five databases, namely Web of Science, Medline, Science Direct, Google Scholar and Scopus. From the search, a total of 253 articles were screened down to 26 articles.

The eligibility criteria were as follows:

- The title and abstract should be relevant to our objective of the review, which is to review the current studies on hospital readmission due to COVID-19.
- The published date for the research should be from 2020 onwards.
- The data collection must include patients diagnosed with COVID-19 *via* reverse transcriptase polymerase chain reaction (RT-PCR).
- The research outcomes must consist of either readmission rate, odds for readmission or risk factor for readmission.
- Case studies reports, news items, review papers, non-English articles are excluded for our review.
- Only articles with full text access are included to ensure the quality of review.

**Figure 1:**
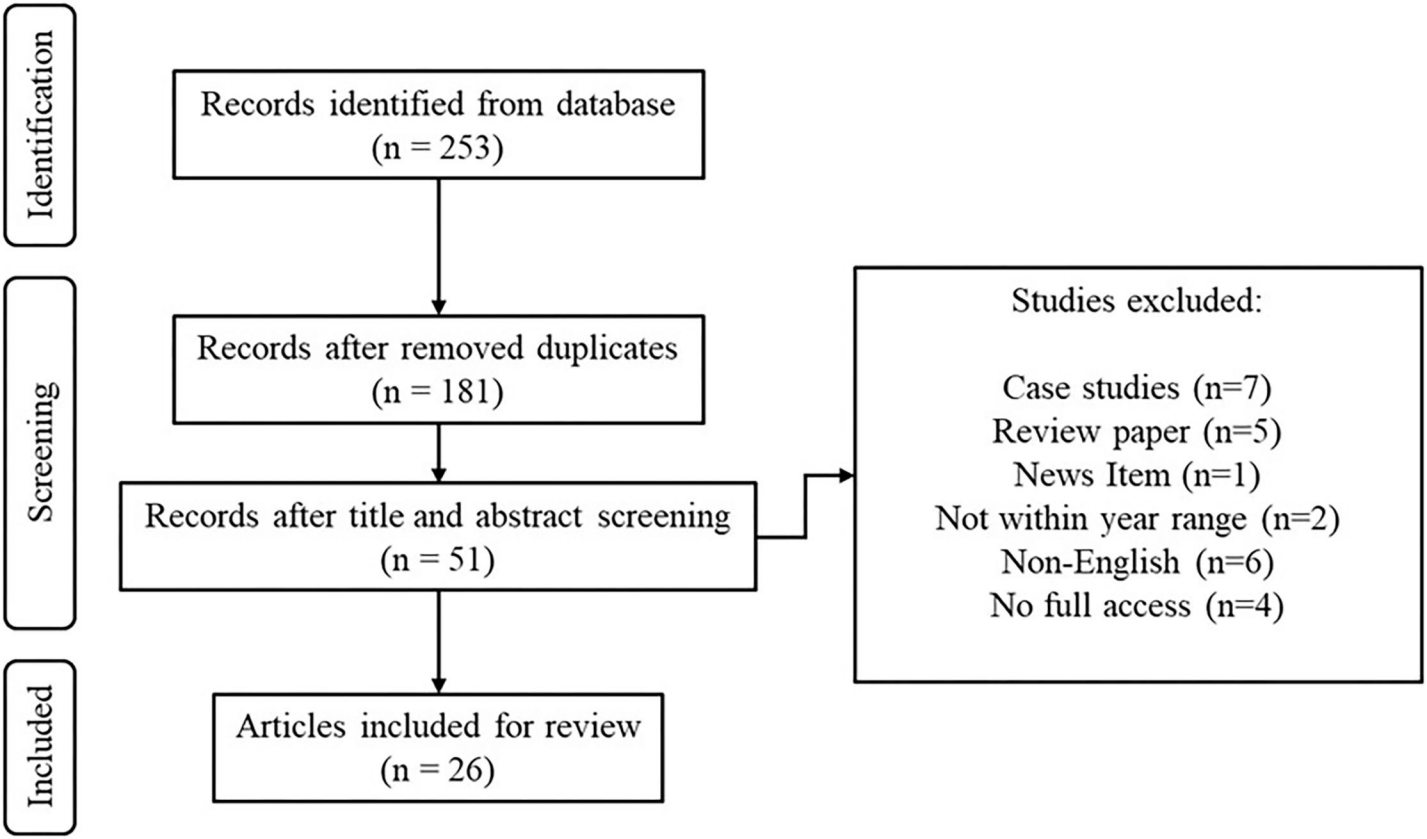
PRISMA diagram for screening of articles from databases.

Figure 1 is the PRISMA diagram for the screening flow of articles from the databases.

### Risk of Bias Assessment

The risk of bias assessment has been carried out based on a few criteria in the Cochrane review, which are:

- Selection Bias. The participants or patient data collected must be readmitted to the hospital due to reinfection of Covid=19.
- Detection Bias. The method of diagnosis for COVID-19 should be the Reverse Transcription Polymerase Chain Reaction (RT-PCR) test.
- Reporting Bias. There should be no missing outcome data in the report.
- Performance Bias and Attrition Bias are not available, as both biases are related to interventional studies, but the review was done based on observational studies.

Each paper is evaluated with the criteria and judged as “Low Risk” (denoted as “+”) if the criteria mentioned are fulfilled, “High Risk” (denoted as “-”) if else, and “Unclear Risk” (denoted as “?”) if not mentioned in the whole report. Table 1 for the risk of bias assessment.

**Table 1:** Risk of bias assessment table.

**References**	**Selection**	**Detection**	**Reporting**	**Risk of bias**
Atalla et al. (8)	+	+	+	Low
Donnelly et al. (3)	+	?	+	Some concerns
Drewett et al. (9)	+	?	+	Some concerns
Green et al. (10)	+	+	+	Low
Guarin et al. (4)	+	+	+	Low
Günster et al. (11)	+	+	+	Low
Gwin et al. (12)	+	?	+	Some concerns
Hong et al. (13)	+	+	+	Low
Iheaku et al. (14)	+	+	+	Low
Jeon et al. (15)	+	+	+	Low
Kirkegaard et al. (16)	+	+	-	Some concerns
Lavery et al. (17)	+	-	-	High
Loerinc et al. (18)	+	+	+	Low
Mehl et al. (19)	+	?	+	Some concerns
Menditto et al. (20)	+	+	+	Low
Mooney et al. (21)	+	?	+	Some concerns
Nematshahi et al. (22)	+	+	-	Some concerns
Parra et al. (23)	+	+	-	Some concerns
Ramos-Martínez et al. (24)	+	+	+	Low
Robinson-Lane et al. (25)	+	+	+	Low
Rodriguez et al. (26)	+	+	+	Low
Taupin et al. (27)	+	+	+	Low
Uyaroglu et al. (28)	+	+	-	Some concerns
Verna et al. (29)	+	?	+	Some concerns
Yang et al. (30)	+	+	-	Some concerns
Yeo et al. (31)	+	+	+	Low

*Green is for “Low Risk”, yellow is for “Unclear Risk”, and red is for “High Risk”*.

## Readmission and Mortality Rate Post COVID-19 Admission

The readmission and mortality rate are important parameters to the medical and healthcare sector such as the hospital administrations. In the COVID-19 pandemic, these parameters cause pressure on the decision and policy makers of healthcare sector.

In this chapter, we will review past studies on the readmission and mortality rate post COVID-19 admission. A total of 23 articles related to readmission and mortality rate post COVID-19 admission are collected for review.

According to research in Germany, the mortality rate was 24.9% (index hospitalization), 23.9% (30-day), 27.9% (90-day), and 29.6% (180-day), with a readmission rate of 26.8%. Günster et al. found that the latter was 52.3% for patients aged >80 years, and the mortality is higher among those who have been ventilated (11).

Next, according to research in England, an 11.3% of readmission rate for 274 individuals was reported. Higher readmission rates are observed among older patients compared to young age groups (17.9 vs. 14.8%, *p* = 0.36), as well as higher mortality in older patients (60.2 vs. 20.3%, *p* <0.001) (21).

Research in South Korea reveals a 4.3% readmission rate with a higher rehospitalization rate for men compared to women (5.5 vs 3.5%). Older age was also the risk factor for a higher readmission rate. Medical benefited pat.ients have a readmission rate of 15.4%, which is higher than patients with health insurance by 4.7 times. (15).

There are nine articles from the United States. Among them, two of the articles used descriptive statistics as their analysis method, six of them used various inferential statistical analysis approaches such as the Chi-square test, Student-*t*-test, Kruskal–Wallis H test, Wilcoxon rank-sum test, Fisher's exact test, and Mann–Whitney U test, and only 1 of them utilizes machine learning in their research. The readmission and mortality rates differ from each other, with the highest readmission rate of 19.9% by Donnelly et al. and the lowest of 4.5% by Yeo et al. For mortality rate, the highest mortality rate is 13% by Robinson-Lane et al., while according to research by Mehl et al., there was no mortality found throughout the research. For the outcomes of the research using machine learning approach, the predictive performance, area under the receiver-operating characteristic curve - readmission is 0.871 (95% CI, 0.830–0.917), while the area under the precision-recall curve – readmission is 0.504 (95% CI, 0.388–0.604) (19).

Two research works have been contributed by China, one of them reported an 8.86% of readmission rate, and 9% for the other one. Both use relatively small data size (79 and 145) and uses inferential statistical approaches in their research, thus the readmission rate has a small difference (0.14%).

There are also two articles from Georgia, with both using descriptive statistics as their analysis approach. According to research with a smaller data size (310), the post-discharge readmission rate was 5.2% with 68.8% of these attributable to COVID-19. The research with a bigger data size (2,399) reveals a 6.4% readmission rate within 30 days. Despite the difference in data size, the result does not have a difference in a big range.

Three research works have been reported from Spain, with one using descriptive statistics, and another two using the inferential statistics method. All three studies show that the readmission rate is around 5% in Spain (4.2%, 4.4%, and 5.4%), One study shows that 0.73% died during the first month post-discharge.

Another research from Iran reveals that the rate of readmission for 30 and 60 days after discharge was 7.6% and 8.1%, respectively, 14.2% readmission rates were reported by Drewett et al. from Australia (9), 9.2% by Green et al. from Israel (10), and 7.1% by Uyaroglu et al. from Turkey (28).

In overall, most of the research focus on readmission rates than mortality rate. On the readmission rate, the lowest is 4.2% by Ramos-Martínez et al. from Spain, and the highest is 19.9% by Donnelly et al. from the United States. Most of the research (13) use the inferential statistical approach in their studies, while only one uses the machine learning approach. The data size ranges from 79 to 126,137. However, there is no specific guide to set the most suitable data size for one research, and all results cannot be compared in terms of accuracy, as all research is regional studies and do not involve data from multi regions.

Table 2 shows the reviews for readmission and/or mortality rate.

**Table 2:** Table of review on readmission and / or mortality rate.

**References**	**Year**	**Data region**	**Data size**	**Statistical analysis**	**Outcomes: Readmission and / or mortality rate**
Günster et al. (11)	2021	Germany	8,769	Descriptive statistics	Median age of 72 years, mortality during index hospitalization = 24.9%, 30-day all-cause mortality rate was 23.9%, the 90-day rate was 27.9%, and the 180-day rate, 29.6%. The latter was 52.3% for patients aged >80 years, 23.6% if not ventilated, during index hospitalization, but 53.0% in case of those ventilated invasively. Readmission rate within 180 days is 26.8%.
Mooney et al. (21)	2021	England	393	Descriptive statistics	11.3% of readmission rate for 274 individuals were reported. Higher readmission rate are observed among older patients compared to young age groups (17.9 vs. 14.8%, *p* = 0.36), as well as higher mortality in older patients (60.2 vs. 20.3%, *p* < 0.001)
Jeon et al. (15)	2020	South Korea	7,590	Descriptive statistics, Chi-square test	Of the 7,590 subjects analyzed in South Korea, there are 4.3% readmission rate with higher rehospitalization rate of men compared to women (5.5 vs. 3.5%). Older age was also the risk factor to higher readmission rate. Medical benefited patients have readmission rate of 15.4%, which is higher than patients with health insurance by 4.7 times.
Yeo et al. (31)	2021	United States	1,062	Descriptive statistics, Chi-square test, Student *t*-test or Mann–Whitney–Wilcoxon nonparametric test	At the end of the study, a total of 48 (4.5%) patients were readmitted within 30 days of discharge, and a median time to readmission was 5 days.
Yang et al. (30)	2021	China	79	Chi-square test, Fisher' *t*-test, Student's *t*-test, and Kruskal-Wallis H test	Readmission rate due to COVID-19 re-positive = 8.86%
Lavery et al. (17)	2020	United States	126,137	Descriptive statistics	Among the 106,543 (85%) surviving patients, 9% (9,504) were readmitted to the same hospital within 2 months of discharge through August 2020.
Rodriguez et al. (26)	2021	United States	2,256 + 855 (validation)	L1-penalized logistic regression (logistic L1), elastic-net logistic regression (logistic EN), and gradient boosted trees (GBT)	The predictive performance: area under the receiver-operating characteristic curve— readmission 0.871 (95% CI, 0.830–0.917); area under the precision-recall curve— readmission 0.504 (95% CI, 0.388–0.604).
Loerinc et al. (18)	2021	Georgia	310	Descriptive statistics	The post-discharge readmission rate was 5.2% with 68.8% of these attributable to COVID-19.
Gwin et al. (12)	2021	United States	151	Two-sided *t*-tests, Wilcoxon rank-sum test, Fisher's exact test	24% hospital return rate was reported, with 11% of revisit rate, 14% of 14-day revisit, and 24% of 30-day revisit rate.
Nematshahi et al. (22)	2021	Iran	416	Descriptive statistics	51 of 416 patients was readmitted. 7.6% of 30-day readmission and 8.1% of 60-day readmission were reported.
Mehl et al. (19)	2021	United States	66	Descriptive statistics	readmission is 6% with no mortality
Ramos-Martínez et al. (24)	2021	Spain	7137	Chi-square test, Fisher's exact test, Student's *t*-test and the Mann-Whitney U Test	4.2% were readmitted. 35 died during hospital readmission (11.7%, *p* = 0.007). 50 died during the first month after discharge (0.73%).
Parra et al. (23)	2020	Spain	1,368	Chi-square test, Fisher's exact test, Student's *t*-test and the Mann-Whitney *U*-Test	4.4% with 74% male who presented COVID-19 were readmitted during the 3 weeks after discharge from hospital
Kirkegaard et al. (16)	2021	Spain	629	Descriptive statistics	Readmission cumulative incidence =5.4% and incidence rate = 0.034 person-years.
Taupin et al. (27)	2021	United States	576	Chi-square test, Fisher's exact test, Wilcoxon rank sum test	76 of 576 COVID-19 hospitalizations resulted in a 30-day revisit (13.2%), including 21 ED visits without admission (3.6%) and 55 readmissions (9.5%).
Robinson-Lane et al. (25)	2021	United States	2,217	Descriptive statistics, Pearson chi-squared test	11.8% rehospitalization was observed, with 55% consists of non-white patients.
Donnelly et al. (3)	2021	United States	1,775	Wilcoxon rank sum tests, Pearson χ2 tests of association	Within 60 days of discharge,19.9% who survived COVID-19 hospitalization were readmitted, 9.1% died, and 27.0% were readmitted or died.
Atalla et al. (8)	2021	United States	339	Pearson's chi-square test, Student *t*-test and Mann-Whitney Wilcoxon test	19/279 were readmitted (6.8%) after a median of 5 days.
Iheaku et al. (14)	2021	Georgia	2,399	Descriptive and observatory	6.4% were readmitted within 30 days. 72% of the readmitted patients were Black or Hispanic
Drewett et al. (9)	2021	Australia	169	Chi-squared and rank sum tests	14.2% were readmitted to hospital within 6 months.
Hong et al. (13)	2021	China	145	Mann–Whitney *U*-test, χ^2^ test	9% of readmission rate with 7 males and 6 females due to repositivity
Green et al. (10)	2021	Israel	618	Descriptive statistics	Of the 544 patient who were discharged, 1.83% died following and 9.2% were re-admitted. Median post discharge follow-up was 59 days (Interquartile range, IQR, 28–161).
Uyaroglu et al. (28)	2021	Turkey	154	Pearson Chi-Square Test or Fisher exact Test, Mann–Whitney *U*-test or Kruskal Wallis Tests	Of 154 patients, 7.1% were readmitted. The median time of readmission was 8.1 days (IQR=5.2).

## Readmission Risk Factors Post COVID-19 Admission

Hospital readmission could be due to different reasons than the main infection (COVID-19), and some clinical factors such as comorbidities may possess a higher risk to the infected person. If the person suffering from certain comorbidity, he may have a higher risk to be readmitted to the hospital post-COVID-19 admission. After a review of the readmission and mortality rate statistics across regions, we will review the readmission risk factors post COVID-19 admission. A total of 18 articles are relevant to this topic.

There are six articles from United States, with three using multivariable logistic regression, while the others use Pearson chi-squared test, Piecewise Cox proportional hazards regression Student *t*-test, and Mann–Whitney Wilcoxon test, respectively. Results wise, Guarin et al. found coronary artery disease and Hispanic ethnicity as the risk factor (4). Age more than, diabetes, CVD, CKD stage 1–5, and CKD stage 5 were reported by Verna et.al (29). Yeo et.al. found that a peak serum creatinine level of ≥1.29 mg/dl during index hospitalization has an odds ratio (OR) of 2.41 (31). Robinson-Lane et al. focus on the ethnicity factor and found that more than half of hospital readmissions within the 60 days following discharge were among non-white patients (55%) (25). Donnelly et al. compared the readmission rate between survivors of COVID-19, pneumonia, and heart failure, and they found that COVID-19 survivors had lower rates of 60-day readmission or death than matched survivors of pneumonia (26.1 vs. 31.7%; *p* = 0.006) and heart failure (27.0% vs. 37.0%; *p* < 0.001) (3). Atalla et al. discovered a few risk factors: hypertension, diabetes, chronic obstructive pulmonary disease (COPD), liver disease, cancer, alcohol, and drug abuse (8).

One research from Italy discovered that cognitive impairment, P/F<300 mmHg, being resident in a geriatric care facility, and neutrophilia as the risk factors contributing to readmission.

Next, according to research from Germany, the risk factors identified are BMI > 40, liver disease, metastatic cancer, and coagulopathy. For age, the OR was 1.08 per year, the odds for increase by a factor of 2.21 per additional 10 years of age (1.082449^∧10^) (11).

The risk factors from research in South Korea are male, 65 years of age or older, having medical benefits, living in Gyeongsangbuk-, prescribed Kaletra, patients with chest radiographs, CT took, and shorter length of stay in the first admission.

According to Nematshahi et al. from Iran, the site of lung involvement. age over 60 years, diabetes, and high creatinine level (≥1.2 mg/dl) are identified as risk factors of readmission (22).

All three study from Spain use logistic regression for their research, and the data sizes are 7,137, 1,368, and 629, respectively. Age, age-adjusted Charlson comorbidity index score, chronic obstructive pulmonary disease (COPD), asthma, hemoglobin level at admission, ground-glass opacification at admission, and glucocorticoid treatment are identified by Ramos-Martínez et al. (24), immunocompromised patients, hypertensive patients, and fever during the 48 h prior to discharge by Parra et al. (23), while prior diagnosis of heart failure, length of stay during index admission >13 days, treatment with corticosteroids and developing pulmonary thromboembolism are identified by Kirkegaard et al. (16).

Diabetes and end stage renal disease are the risk factors from the research in Georgia. Increased length of stay during the index admission, ICU admission, supplemental oxygen and high flow nasal oxygen (HFNO), and chronic obstructive respiratory disease (COPD) are the risk factors based on research from Australia. Hong et al. from China also had research and found that longer virus shedding duration, and lower IgG levels as the risk factors for readmission (13).

On the other hand, Green et al. from Israel found that older age, age above 80 years, higher CCI, and solid organ transplantation as the risk factors (10). Individual comorbidities included in CCI are the history of coronary artery disease, CVA, diabetes mellitus, severe kidney disease metastatic disease, and higher systolic blood pressure.

Lastly, research in Turkey reveals malignancy and hypertension as the risk factors for readmission (28).

**Figure 2:**
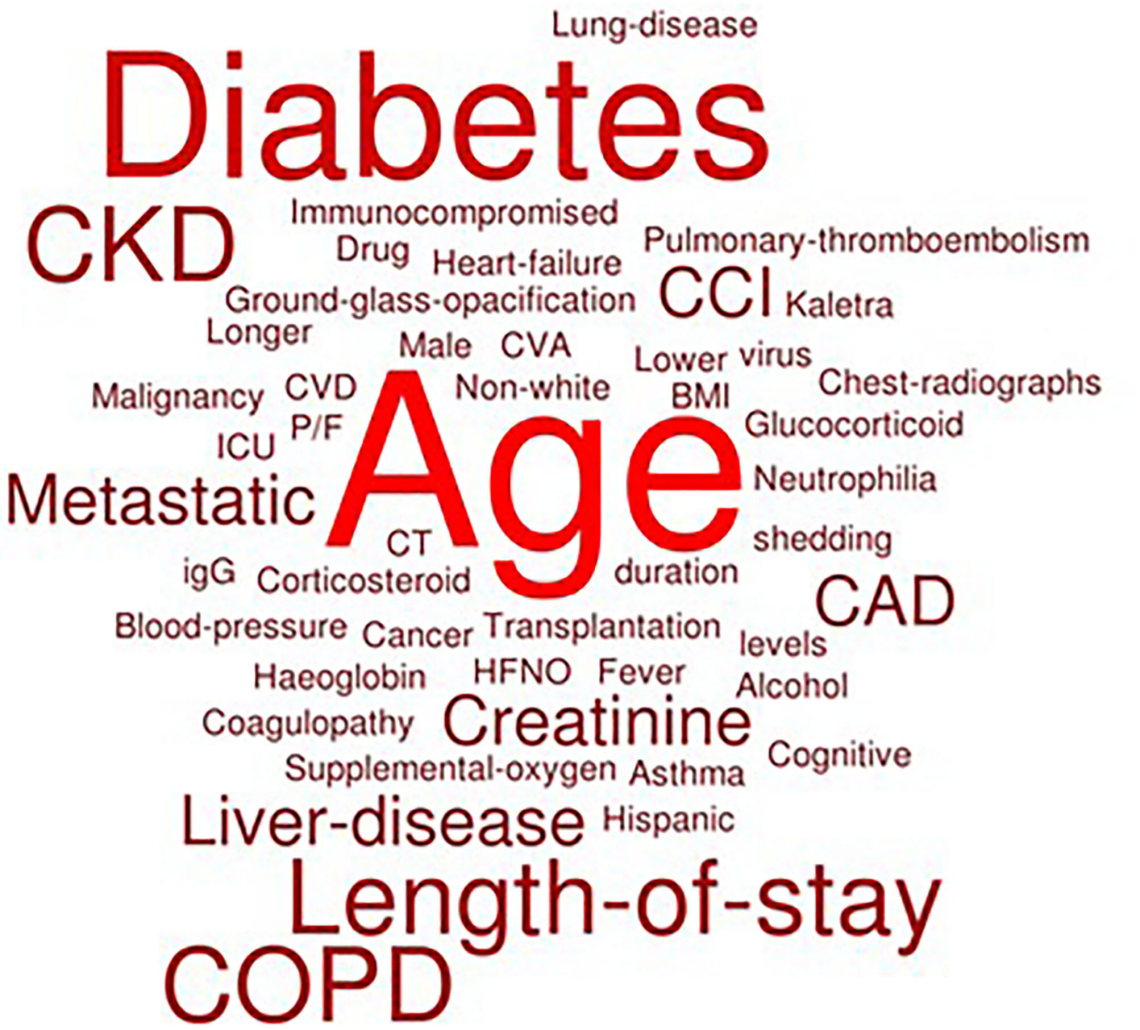
Word cloud for the risk factors of readmission.

A word cloud is constructed based on the findings by extracting the risk factors from all articles into the system. If the frequency of risk factors is higher, then the word size will be bigger. Figure 2 is a word cloud to summarize and visualize the risk factors mentioned in this chapter.

Overall, the logistic regression is prevalent in the research on risk factors of readmission post-COVID-19 admission, despite each of the research coming out with different outcomes. From the word cloud, age is the most dominant risk factor for readmission, followed by diabetes, high length of stay, COPD, CKD, liver disease, metastatic disease, and CAD.

Table 3 shows the reviews for general readmission risk factors.

**Table 3:** Table of review on general readmission risk factors post COVID-19 admission.

**References**	**Year**	**Data region**	**Data size**	**Statistical analysis**	**Outcomes: Risk factors of readmission**
Guarin et al. (4)	2021	United States	275	Multivariable logistic regression	Coronary artery disease (CAD) odds ratio (OR), 2.15 (95% confidence interval [CI]: 1.04–4.44; *p* = 0.039), Hispanic ethnicity OR, 3.16 (95% CI: 1.01–9.88; *p* = 0.048).
Verna et al. (29)	2021	United States	29,659	Multivariable logistic regression	Age >60 vs. 18–40 (odds ratio [OR] = 1.92, 95% confidence interval [CI] = 1.48, 2.50), admitted in the Northeast vs. West (OR = 1.43, 95% CI = 1.14, 1.79) or South (OR = 1.28, 95% CI = 1.11, 1.49), Diabetes (OR=1.34, 95% CI = 1.12, 1.60), CVD (OR = 1.46, 95% CI = 1.23, 1.72), CKD stage 1–5 (OR = 1.51, 95% CI = 1.25,1.81) and CKD stage 5 (OR = 2.27, 95% CI = 1.81, 2.86)
Menditto et al. (20)	2021	Italy	283	Welch's *t*-test, Mann Whitney U test	Cognitive impairment (OR 17.3 [CI 4.7–63.2]), P/F <300 mmHg (OR 8.6 [CI 1.6–44.3]), Being resident in geriatric care facility (OR 7.6 [CI 2.1–26.4]) and Neutrophilia (OR 5.8 [CI 1.6–22.0]).
Günster et al. (11)	2021	Germany	8,769	Multivariable logistic regression, Wald test, squared Pearson correlation (R^2^)	BMI > 40 (OR 2.01, 95%-CI 1.33 to 3.05), Liver disease (OR 2.45, 95%-CI 1.85–3.25), Metastatic cancer (OR 8.02, 95%-CI 3.57–18.00), and Coagulopathy (OR 2.31, 95%-CI 1.82–2.94). For age, the OR was 1.08 per year, the odds for increase by the factor 2.21 per additional 10 years of age (1.082449^∧^10)
Jeon et al. (15)	2020	South Korea	7,590	Logistic regression	Male (Odds Ratio (OR): 1.340, 95% CI: 1.055,1.706), 65 years of age or older (OR: 2.235, 95% CI: 1.111–4.497), Having medical benefits (OR: 2.757, 95% CI: 2.040–3.725), and Living in Gyeongsangbuk-do (OR: 2.876, 95% CI: 2.144–3.857), Prescribed Kaletra (OR: 1.388, 95% CI:1.056–1.827), Patients with chest radiographs (OR: 1.591, 95% CI: 1.121–2.258) and CT taken (OR: 1.330, 95% CI: 1.031–1.71), Shorter length of stay in first admission (OR: 0.945, 95% CI: 0.933–0.958).
Yeo et al. (31)	2021	United States	1,062	Multivariable logistic regression	Peak serum creatinine level of ≥1.29 mg/dL during the index hospitalization, vs. creatinine of <1.29 mg/dL, (adjusted odds ratio: 2.41; 95% CI: 1.23–4.74).
Nematshahi et al. (22)	2021	Iran	416	Regression model	Site of Lung involvement (OR > 4). Age over 60 years (OR = 1.12), underlying disease especially Diabetes (OR = 3.43), high creatinine level (≥1.2 mg/dl) (OR = 2.15)
Ramos-Martínez et al. (24)	2021	Spain	7,137	Univariate and Multivariate logistic regression	Age [odds ratio (OR): 1.02; 95% confident interval (95% CI): 1.01–1.03], age-adjusted Charlson comorbidity index score (OR: 1.13; 95% CI: 1.06–1.21), chronic obstructive pulmonary disease (OR: 1.84; 95% CI: 1.26–2.69), asthma (OR: 1.52; 95% CI: 1.04–2.22), hemoglobin level at admission (OR: 0.92; 95% CI: 0.86–0.99), Ground-glass opacification at admission (OR: 0.86; 95% CI:0.76–0.98) and Glucocorticoid treatment (OR: 1.29; 95% CI: 1.00–1.66)
Parra et al. (23)	2020	Spain	1,368	Univariate and Multivariate logistic regression	Immunocompromised patients (*N* = 10.2%) (*p* = 0.04), Hypertensive patients (*p* = 0.07), Fever during the 48 h prior to Discharge (*p* < 0.001)
Kirkegaard et al. (16)	2021	Spain	629	Multivariate logistic regression	Prior diagnosis of heart failure (OR 4.09; 95% CI 1.35–12.46; *p* = 0.013), length of stay during index admission >13 days (OR 2.72; 95% CI 1.21–6.12; *p* = 0.015), treatment with corticosteroids (OR 2.39; 95% CI 1.01–5.70; *p* = 0.049) and developing pulmonary thromboembolism (OR 11.59; 95% CI 2.89–46.48; *p* = 0.001)
Robinson-Lane et al. (25)	2021	United States	2,217	Analysis of variance and Pearson chi-squared test	Black patients have the lowest physician follow-up post discharge (*n* = 65, 60.2%) and the longest delays in returning to work (average 35.5 days). More than half of hospital readmissions within the 60 days following discharge were among non-white patients (*n* = 144, 55%). The most post discharge deaths are white patients (*n* = 153, 21.5%)
Donnelly et al. (3)	2021	United States	1,775	Piecewise Cox proportional hazards regression	COVID-19 survivors had lower rates of 60-day readmission or death than matched survivors of pneumonia (26.1 vs. 31.7%; *p* = 0.006) and heart failure (27.0 vs. 37.0%; *p* < 0.001). However, COVID-19 survivors had higher rates of readmission or death within the first 10 days after discharge than matched survivors of pneumonia (13.4 vs. 9.7%; *p* = 0.01) and heart failure (13.9 vs. 8.8%; *p* < 0.001)
Atalla et al. (8)	2021	United States	339	Pearson's chi-square test, Student *t*-test and Mann-Whitney Wilcoxon test	Hypertension (68.4% *vs*. 44.1%, *p* = 0.038), diabetes (57.9 vs. 32.2%, *p* = 0.021), chronic pulmonary disease (57.9 vs. 12.8%, *p* < 0.001), liver disease (15.8 vs. 2.5%, *p* = 0.001), cancer (21.1 vs. 7.2%, *p* = 0.03), alcohol (26.3 vs.4.1%, *p* < 0.001), and drug abuse (15.8 vs. 4.4%, *p* = 0.027) were more common among patients who readmitted, patients readmitted had a lower ICU utilization (10.5 vs. 34.4%, *p* = 0.032) and intubation rate (0 vs. 20%, *p* = 0.03) during index hospitalization.
Iheaku et al. (14)	2021	Georgia	2,399	Descriptive and observatory	Diabetes (*p*: 0.03) and end stage renal disease (*p*: 0.004)
Drewett et al. (9)	2021	Australia	169	Chi-squared and rank sum tests	Increased length of stay during index admission (5 vs. 7 days, *p* = 0.04), ICU admission (*p* = 0.04), supplemental oxygen (*p* = 0.03) and high flow nasal oxygen (HFNO) (*p* = 0.01), chronic respiratory disease (21.7 vs. 41.7%, *p* = 0.12)
Hong et al. (13)	2021	China	145	Covariate binary logistic regression analysis with forward conditional method	Longer virus shedding duration [odd ratio (OR) = 1.280, 95% confidence interval (CI):1.052–1.558; *p* = 0.013], and lower IgG levels (OR = 0.690, 95% CI: 0.528–0.901; *p* = 0.007]
Green et al. (10)	2021	Israel	618	Cox proportional hazards model; with the Fine and Gray methodology, multiple regression model	Older age (HR 1.03 for every year, 95% CI 1.01–1.04, *p* = 0.002); age above 80 years (HR4.93, 95% CI 1.14–21.29, *p* = 0.032; and higher CCI (HR for every point 1.34, 95% CI 1.24–1.44, *p* < 0.0001). Individual comorbidities included in CCI: history of coronary artery disease, CVA, diabetes mellitus, severe kidney disease metastatic disease and a higher systolic blood pressure. Solid organ transplantation (HR 4.97, 95% CI 2.09–11.83, *p* = 0.0009). In multivariate analysis: solid organ transplantation (HR 3.37, 95% CI 2.73–7.5, *p* = 0.0028) and CCI (HR 1.34 for every point, 95% CI 1.23–1.46, *p* < 0.0001)
Uyaroglu et al. (28)	2021	Turkey	154	Pearson Chi-Square Test or Fisher exact Test, Mann-Whitney *U*-test or Kruskal Wallis Tests	Malignancy (18.7 vs. 2.1%, *p* = 0.04) and hypertension (45.5 vs. 14%, *p* = 0.02)

## Discussions

In this chapter, we will discuss the limitations, research gaps, and potential future directions of research on COVID-19 readmissions.

Most of the research involves data collection from a single center, which is focused on one specific region. As we all know, COVID-19 is a widespread disease that affects public health globally, if the study focuses on one single region, the research insights may be valuable only to the people of the same region, whereas outside institutions might not use those insights as reference. To make the research outcomes practical and applicable to most regions, comprehensive data collection should be made across regions.

Next, the study duration of research is to be questioned. COVID-19 is a rapid-evolving phenomenon, delta and omicron variants have emerged lately, and this will impact the accuracy of results if the study was done before the emergence of the variants. Research on COVID-19 should be carried out progressively and continuously in a long term to obtain updated info and insights from time to time. However, if the study duration is too long, the insights may not be relevant anymore if the publishing is after the lengthy study duration. The timeline of the research and experiments should be planned carefully to make sure the insights obtained are not outdated.

Most of the experimental designs do not include a control group for results comparison. A control group is important to allow researchers to confirm that the study results are due to the manipulation of independent variables (infection of COVID-19) rather than extraneous variables. The control group is the group of participants who are not exposed to the manipulated variable and the results are to be compared with the groups exposed to the manipulated variable.

As one of the future directions, the machine learning approach can be utilized in the statistical analysis of readmission and mortality due to COVID-19. As reviewed in Chapter 2, most of the studies only used inferential statistical analysis. Machine Learning (ML) is a subfield of computer science and artificial intelligence. ML deals with building systems (algorithms, models) that can learn from data and observations, instead of explicitly programmed instructions and rules. Machine learning finds generalizable patterns from data and can make predictions based on the data, while inferential statistics are used to formalize the relationships between variables. Machine learning can be applied in readmission studies in order to predict the readmission rate according to each patient's condition, including comorbidities, and demographic and geographic factors. Also, machine learning can be used in the prognosis of the potential rise in certain regions with available outcomes as reference. The insight from the inferential statistics is insufficient for healthcare policies and decisions, however, with predictive insights from machine learning, the healthcare sector can make and change decisions to prevent or curb the phenomenon.

Next, from Chapter 3, we can notice a few dominant risk factors of readmission being identified, such as age, diabetes, and COPD. One potential direction is to investigate the relationship and make predictions of hospital readmissions on age factors. Studying the influence of age on the risk of readmission could help healthcare personnel to give more attention to the aged patients while designing interventions, especially for old folks could tackle the dominant risk factor and reduce the readmission rates. The same goes for diabetes, COPD, and other risk factors, more research could be done specifically on the certain risk factor, or interventional studies could be designed to tackle and curb the COVID-19 readmission.

Apart from the conventional risk factors which increase the risk of readmission, interestingly, the rate of readmission increases significantly in patients with dysbiotic gastrointestinal symptoms, according to a gastrointestinal study in Istanbul (32). Also, the study found that intestinal microbiota affects disease morbidity and mortality (33). In particular, the Th17/II17-116 immune cascade is responsible for COVID-19 pathology and can increase the severity of the disease (34). From the evidence, gastrointestinal symptoms may be a new direction of research as a COVID-19 readmission risk factor. Other symptoms may also be the focus of research, for instance, respiratory symptoms, which are not yet widely explored.

Also, no study has been reported with a collection of data involving multi regions. Despite the collection, the process will be harder, and the data will be harder in synchronization, the research outcomes will be more useful and practical to the public. As we all know, COVID-19 is spreading worldwide, and study from a single center only provides useful insights for that particular region.

## Conclusion

To summarize, studies on readmission and mortality of COVID-19 had been done in various regions and promising statistics have been obtained. The readmission and mortality rates of COVID-19 and readmission risk factors of COVID-19 have been reviewed and discussed. Despite a number of research work has been done, there are a few limitations and research gaps to focus on and improve in future research. A few future research directions have been proposed, including the utilization of machine learning in statistical analysis, investigation of dominant risk factors, experimental design on interventions to curb dominant risk factors and increasing the scale of data collection from single centered to multi centered. COVID-19 is a rapidly evolving phenomenon, and all research should be done progressively and continuously in a fast manner so that the research insights are relevant and updated from time to time.

## Data Availability Statement

The original contributions presented in the study are included in the article/supplementary material, further inquiries can be directed to the corresponding authors.

## Author Contributions

All authors contributed equally to design, analyze, and drafting this manuscript. All authors contributed to the article and approved the submitted version.

## Funding

This study was supported in parts by the 2020 APT EBC-C (Extra-Budgetary Contributions from China) Project on Promoting the Use of ICT for Achievement of Sustainable Development Goals, and Association of Commonwealth University (ACU) United Kingdom, and Universiti Malaya under Grant IF015-2021 and IF063-2021.

## Conflict of Interest

The authors declare that the research was conducted in the absence of any commercial or financial relationships that could be construed as a potential conflict of interest.

## Publisher's Note

All claims expressed in this article are solely those of the authors and do not necessarily represent those of their affiliated organizations, or those of the publisher, the editors and the reviewers. Any product that may be evaluated in this article, or claim that may be made by its manufacturer, is not guaranteed or endorsed by the publisher.
